# Xanthohumol alleviates oxidative stress and impaired autophagy in experimental severe acute pancreatitis through inhibition of AKT/mTOR

**DOI:** 10.3389/fphar.2023.1105726

**Published:** 2023-01-18

**Authors:** Yaru Huangfu, Xiuxian Yu, Chengyu Wan, Yuda Zhu, Zeliang Wei, Fan Li, Yilan Wang, Kun Zhang, Shiyi Li, Yuman Dong, Yangying Li, Hai Niu, Guang Xin, Wen Huang

**Affiliations:** Laboratory of Ethnopharmacology, Tissue-orientated Property of Chinese Medicine Key Laboratory of Sichuan Province, West China School of Medicine, West China Hospital, Sichuan University, Chengdu, China

**Keywords:** acute pancreatitis, xanthohumol, autophagy, inflammation, oxidative stress

## Abstract

Severe acute pancreatitis (SAP) is a lethal gastrointestinal disorder, yet no specific and effective treatment is available. Its pathogenesis involves inflammatory cascade, oxidative stress, and autophagy dysfunction. Xanthohumol (Xn) displays various medicinal properties,

including anti-inflammation, antioxidative, and enhancing autophagic flux. However, it is unclear whether Xn inhibits SAP. This study investigated the efficacy of Xn on sodium taurocholate (NaT)-induced SAP (NaT-SAP) *in vitro* and *in vivo*. First, Xn attenuated biochemical and histopathological responses in NaT-SAP mice. And Xn reduced NaT-induced necrosis, inflammation, oxidative stress, and autophagy impairment. The mTOR activator MHY1485 and the AKT activator SC79 partly reversed the treatment effect of Xn. Overall, this is an innovative study to identify that Xn improved pancreatic injury by enhancing autophagic flux *via* inhibition of AKT/mTOR. Xn is expected to become a novel SAP therapeutic agent.

## 1 Introduction

Acute pancreatitis refers to an inflammatory condition in which the pancreas develops necrosis and tissue lesions (Hines and Pandol, 2019). In mild cases, the disease involves only the pancreas and resolves spontaneously. While, around 20% of cases will progress rapidly to severe acute pancreatitis (SAP) with a poor prognosis and a mortality rate as high as 30% (Banks et al., 2013). Current therapeutic agents, however, cannot alter the course of the disease (Mederos et al., 2021). Therefore, developing drugs that target the mechanisms of disease is the priority.

Autophagy is the primary cellular pathway for organelle, lipid, and long-lived protein degradation and recycling (Yao et al., 2021). SAP is characterized by impaired autophagy due to damaged lysosomes and abnormal autophagosome formation (Habtezion et al., 2019). According to recent studies, the pancreatic specific knockout of autophagy-related proteins in mice led to spontaneous pancreatitis and enhancing autophagy by administration of trehalose alleviates arginine-induced SAP (Diakopoulos et al., 2015; Biczo et al., 2018). Autophagy prevents damage caused by reactive oxygen species (ROS) by clearing damaged organelles. Oxidative stress caused by impaired autophagy can promote inflammatory responses. And, dysregulated autophagy alone promotes the inflammatory response (Gukovskaya et al., 2017). Cross talk interacts between autophagy, oxidative stress, and inflammation. However, it is unknown if inflammatory mediators, such as cytokines, influence autophagy during pancreatitis pathogenesis.

Serum interleukin-17 (IL-17) concentrations in SAP patients correlate with disease severity, making it a novel indicator with predictive value (Dai et al., 2015; Li et al., 2021). Interestingly, IL-17 affects autophagy differently depending on the cell type. In liver epithelia and lung epithelia, IL-17 inhibits autophagy, while in B cells and RAW macrophages, it induces autophagy (Liu et al., 2013; Yuan et al., 2014; Orosz et al., 2016; Zhou et al., 2016). On pancreatic acinar cells, no study has linked IL-17 to autophagy to date. Together, inflammatory cascades, oxidative stress, and autophagy dysfunction are all factors contributing to the pathogenesis of SAP. Attenuating SAP might be accomplished by reducing inflammatory and oxidative stress as well as restoring impaired pancreatic autophagy.

Xanthohumol (Xn), a naturally occuring prenylated chalcone compound isolated from hops (the female inflorescences of *Humulus lupulus* L.), has been drawn in significant consideration because of its medicinal properties, including anti-oxidant activities, anti-inflammation, and enhancing autophagic flux (Lv et al., 2017; Sun et al., 2021). Furthermore, late reports revealed that Xn activates autophagy signaling pathways *via* inhibiting AKT/mTOR activity (Sun et al., 2021). The protective effect of Xn on SAP, however, has not been studied.

Collectively, this study investigated the efficacy and potential mechanisms of Xn against sodium taurocholate (NaT)-induced SAP (NaT-SAP). Xn restored autophagic flux and suppressed the oxidative stress *via* inhibiting AKT/mTOR. Moreover, we firstly found Xn alleviated the serum levels of IL-17 on NaT-SAP. According to our current research, SAP may benefit from Xn as a new treatment option.

## 2 Materials and methods

### 2.1 Reagents

**FIGURE 1:**
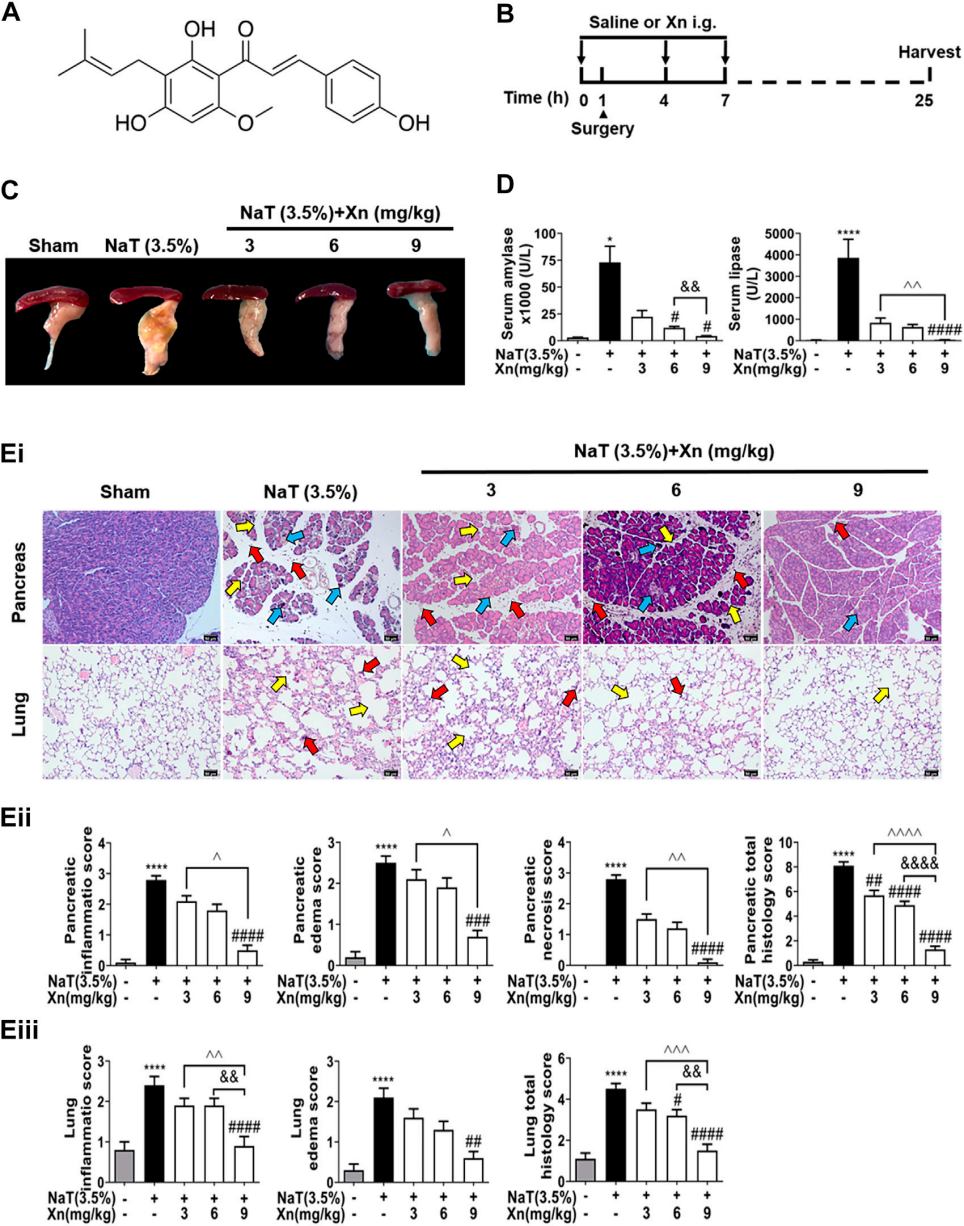
Xn attenuates biochemical and histopathological responses in NaT-SAP mice. **(A)** The chemical structure of Xn. **(B)** Pancreatitis induction and Xn administration scheme. **(C)** Typical images of freshly harvested pancreatic tissues. **(D)** Serum amylase and lipase activity. **(Ei)** Pancreatic and pulmonary H&E images. The red arrow points to inflammation, the yellow arrow points to edema, and the blue arrow points to necrosis. **(Eii)** Histopathological scores of pancreatic tissues. **(Eiii)** Histopathological scores of lung tissues. (mean ± SD; *n* = 4) ^∗^ *p* < 0.05 and ^∗∗∗∗^ *p* < 0.0001 *versus* Sham. ^#^ *p* < 0.05, ^##^ *p* < 0.01, ^###^ *p* < 0.001 and ^####^ *p* < 0.0001 *versus* NaT. ^&&^ *p* < 0.01, and ^&&&&^ *p* < 0.0001 *versus* Xn (6 mg/kg). ^*p* < 0.05, ^^*p* < 0.01, ^^^*p* < 0.001 and ^^^^*p* < 0.0001 *versus* Xn (9 mg/kg).

Purified Xn (# 6754-58-1, HPLC analysis ≥99%, C_21_H_22_O_5_, MW: 354.40) was from Bide Pharmaceutical Technology Co., Ltd. (# BD156629, Shanghai, China) (chemical structure shown in Figure 1A). NaT (# 86339), Collagenase IV (# C4-BIOC), Triton X-100 (# X100) were from Sigma-Aldrich (Saint Louis, United States). Hoechst 33342 (# 62249) and propidium iodide (PI; # P1304MP) were from Molecular Probes (Eugene, United States). Lactase dehydrogenase (LDH)-Glo™ Cytotoxicity Assay Kit (# J2380) was provided by Promega Corporation (Madison, United States). Protease and phosphatase inhibitors (# P1045), RIPA (# P0013C), IL-6 ELISA kit (# PI326), IL-10 ELISA kit (# PI522), IL-17 ELISA kit (# PI545), TNF-α ELISA kit (#PT512), malondialdehyde (MDA) activity assay kit (# S0131S), superoxide dismutase (SOD) activity assay kit (# S0103) and 2′,7′-dichlorodihydrofluorescein diacetate (DCFH-DA; # S0033S) were all from Beyotime Biotechnology (Shanghai, China). The following antibodies were used: Nrf2 (# bs-1074R), p-P65(# bs-3543R), P65(# bs-23217R) were from Bioss (Beijing, China); HO-1 (# 43966), p-AKT (# 5012), p-mTOR (# 5536), LC3B (# 3836), β-actin (# 58169), GAPDH (# 5174) were from Cell Signaling Technology (Danvers, United States). P62 (# A11483) was from ABclonal Technology (Wuhan, China). SC79 (# HY-18749) and MHY1485 (# HY-B0795) were from MedChemExpress (South Brunswick, United States).

### 2.2 Ethics and animals

We conducted all animal-related experiments with the approval of the Animal Ethics Committee of West China Hospital, Sichuan University (No. 20211164A). Approximately 25–30 g male Balb/C mice (between 6 and 8 weeks old) were obtained from Dashuo Experimental Animal Co. Ltd (Chengdu, China) and fed under SPF-condition (20°C–22°C, a relative humidity of 55%, 12 h of light and dark). Food and water were freely accessible to all mice.

### 2.3 Animal model of SAP and treatments

Groups were randomly assigned: sham-operated (Sham) group, NaT-SAP group, and Xn (3, 6, or 9 mg/kg.BW) + NaT groups. The NaT-SAP models were established as described previously (Perides et al., 2010). Briefly, after gently taking out the duodenum from deep in the wound, the duodenum was rotated to reveal the biliary biliopancreatic duct and periampullary region. Then, the cannula was passed through a small hole that has been created in the duodenal wall directly opposite to the papilla. Next, retrograde injected the pancreatic duct with 3.5% NaT (100  μL min^−1^) by infusion pump. In the intervention groups, mice were treated with intragastric administration of Xn (3, 6, or 9 mg/kg.BW) for 1 h before and 3 h and 7 h after modeling (Figure 1B). Mice were euthanized by the cervical dislocation method at 24 h after modeling.

### 2.4 Histopathology

Immediately after the lung and pancreas tissues are removed, they were fixed with 10% formalin, dehydrated in ascending alcohol solutions (30%, 50%, and 70%), embedded by paraffin. Following that, the wax blocks were cut into 5 μm slices. Slices stained with hematoxylin and eosin (H&E) were viewed by the microscope (AX10 imager A2/AX10 cam HRC, Zeiss, Germany). Pancreatic and lung injury were scored according to Wildi S et al. (Wildi et al., 2007) (inflammation, edema, and necrosis; from 0 to 3) and Osman et al. (Osman et al., 1998) (inflammation, and edema; from 0 to 3), respectively.

### 2.5 Immunohistochemical

Prepared pancreatic sections were for immunohistochemical staining as described previously (Liu et al., 2014). Briefly, after being deparaffinized and rehydrated, for quenching endogenous peroxidase, incubation of the samples in 3% H2O2 solution in methanol was performed (10 min, room temperature (RT)). Citrate buffer was used to retrieve antigens. Then slices were pre-blocked with normal goat serum (RT, 10 min), and then incubated with anti-P62 (1:100 dilution, 12h, 4°C), followed by biotinylated secondary antibodies (1:500 dilution, RT, 30 min). For coloring, 3, 3-diaminobenzidine substrate solution was added for 5 min, followed by washing with PBS. Following hematoxylin staining, the sections were counterstained and then imaged using the microscope (BA400Digital, MOTIC, China). Analyzing the images was done with Image-Pro Plus 6.0 (Media Cybernetics, MD, United States).

### 2.6 Serum amylase and lipase detection

Blood samples were centrifuged (1000 × g, 10 min). Serum (diluted with 0.9% saline solution in a ratio of 1:4) was for lipase and amylase measurement by automatic biochemical analyzer (Cobas 8000, Roche, Switzerland).

### 2.7 Isolation of primary pancreatic acinar cells

Mice primary pancreatic acinar cells isolation was performed as previously described (Gerasimenko et al., 2013). Briefly, the fresh pancreas was rapidly collected after euthanizing the mice under sterile conditions. A 19-min digestion at 37°C with collagenase IV (200 U/ml) was performed on the rinsed pancreas. A 100 mm cell strainer was used to isolate cells from pancreatic fragments mechanically dissociated. The filtrate was centrifuged (700 rpm, 2 min) to obtain cell pellets. Resuspend and wash the pellet twice. And then extracellular solution (pH 7.35; HEPES) was used to resuspended the cells.

### 2.8 Detection of necrotic cell death

After indicated treatments and double stained with Hoechst 33342 (50 μg/ml) and PI (1 μmol/ml) for 10 min at RT, acinar cells were fixed on slides (Cosker et al., 2014). Automatic fluorescence microscopes (Axio Imager Z2, Zeiss, Germany) was used to capture images of fixed cells on slides. Based on PI-positive cells/Hoechst 33342-stained cells ratio, necrotic cells were calculated as a percentage.

### 2.9 LDH release assay

The cellular injury was determined by LDH leakage using a kit based on the bioluminescent method. After indicated treatment, acinar cells were centrifugated (300 × g, 30 s) to collect the supernatants. The cells were lysed using 0.2% Triton X-100 for total LDH samples. 50 μl of fresh detection reagent was added to an equal volume of sample. After 60-min incubation at RT during darkness, the microplate reader (Synergy H1, BioTek, United States) was used for the luminescence detection. The percentage of LDH release was calculated as follows: (LDH release from acinar cells minus medium background) divided by (total LDH minus medium background).

### 2.10 Detection of TNF-α, IL-6, IL-10 and IL-17 levels

Serum cytokines were detected after blood samples were centrifuged (1000 × g, 10 min). After indicated treatments, 0.2% Triton X-100 was added into acinar cells for cytokines measurement *in vitro*. The supernatants were collected after centrifugation (1000 × g, 3 min). The cytokines in serum and pancreatic acinar cell lysates were determined by the employment of ELISA kits.

### 2.11 Detection of cytosolic ROS

Cytosolic ROS was detected using DCFH-DA. Acinar cells (after indicated treatments for 50 min) were incubated in PBS with DCFH-DA (10 μM, 37°C, 20 min) during darkness. After washing, nuclear DNA was labeled with Hoechst 33342 (50 μg/ml). A confocal microscope (N-SIM S, Nikon, Japan) was used to capture images of fixed cells on slides.

### 2.12 Detection of pancreatic levels and SOD activities

Fresh pancreatic tissues and acinar cells were homogenized in PBS and centrifuged (15,000 × g, 10 min) for supernatants. We evaluated the MDA levels and SOD activities in supernatants using colorimetric assay kits.

### 2.13 Western blot analysis

An aliquot of 20 μg of total protein was loaded on 10% or 15% SDS-PAGE gel and transferred onto PVDF membranes (IPFL00010, Millipore, United States). At RT, membranes were blocked for 1 h with 5% nonfat milk and then probed with primary antibodies for 1 h. Membranes were incubated with the secondary antibody conjugated with horseradish peroxidase and identified by an enhanced chemiluminescence detection system (P0018S, Beyotime Biotechnology, China). Protein intensities were quantified using Fiji software and normalized to the intensities of the corresponding β-actin or GAPDH as loading controls (Schindelin et al., 2012).

### 2.14 Statistical analysis

Data are expressed as means ± SD. For multiple comparisons, one-way ANOVA was used followed by Dunnett’s post-hoc test with Prism 8.0 software (GraphPad Software Inc., La Jolla, CA, United States). Statistical significance was defined as *p* < 0.05.

### 2.15 Molecular docking

The 2D structures of Xn were downloaded from the PubChem database (https://pubchem.ncbi.nlm.nih.gov/) and the 3D structures of PI3K were screened from the RCSB Protein Data Bank (https://www.rcsb.org/). We used AutoDock Tools (Version 1.5.6) and AutoDock Vina (Version 1.1.2) to dock Xn with the target and calculate the free binding energies. After docking, PyMOL (version 2.3) was used to visualize the interactions and binding modes between the molecule and target.

## 3 Results

### 3.1 Xn concentration-dependently protected against NaT-SAP in mice

We investigated the efficacy of Xn on NaT-SAP mice model, which is a representative model of acute biliary pancreatitis (Lerch and Gorelick, 2013). NaT-SAP led to a sharp increase in pancreatic injury, as measured by inflammatory infiltration, oedema, and necrosis using histopathology score. However, Xn treatment concentration-dependently reduced pancreatic injury (Figures 1C–Ei, Eii). In SAP, acute lung injury ranks among the most critical distant organ injuries (Ge et al., 2020). We then examined the efficacy of Xn on SAP associated lung injury by histopathology score. As expected, the NaT group had higher lung histopathological scores than the Sham group. However, Xn reduced lung injury concentration-dependently (Figure 1Eiii). Meanwhile, NaT-SAP resulted in excessive serum amylase and lipase activity. However, Xn treatment group showed lower of these compared to the NaT group (Figure 1D).

### 3.2 Xn concentration-dependently protected against NaT-induced necrosis and inflammation

**FIGURE 2:**
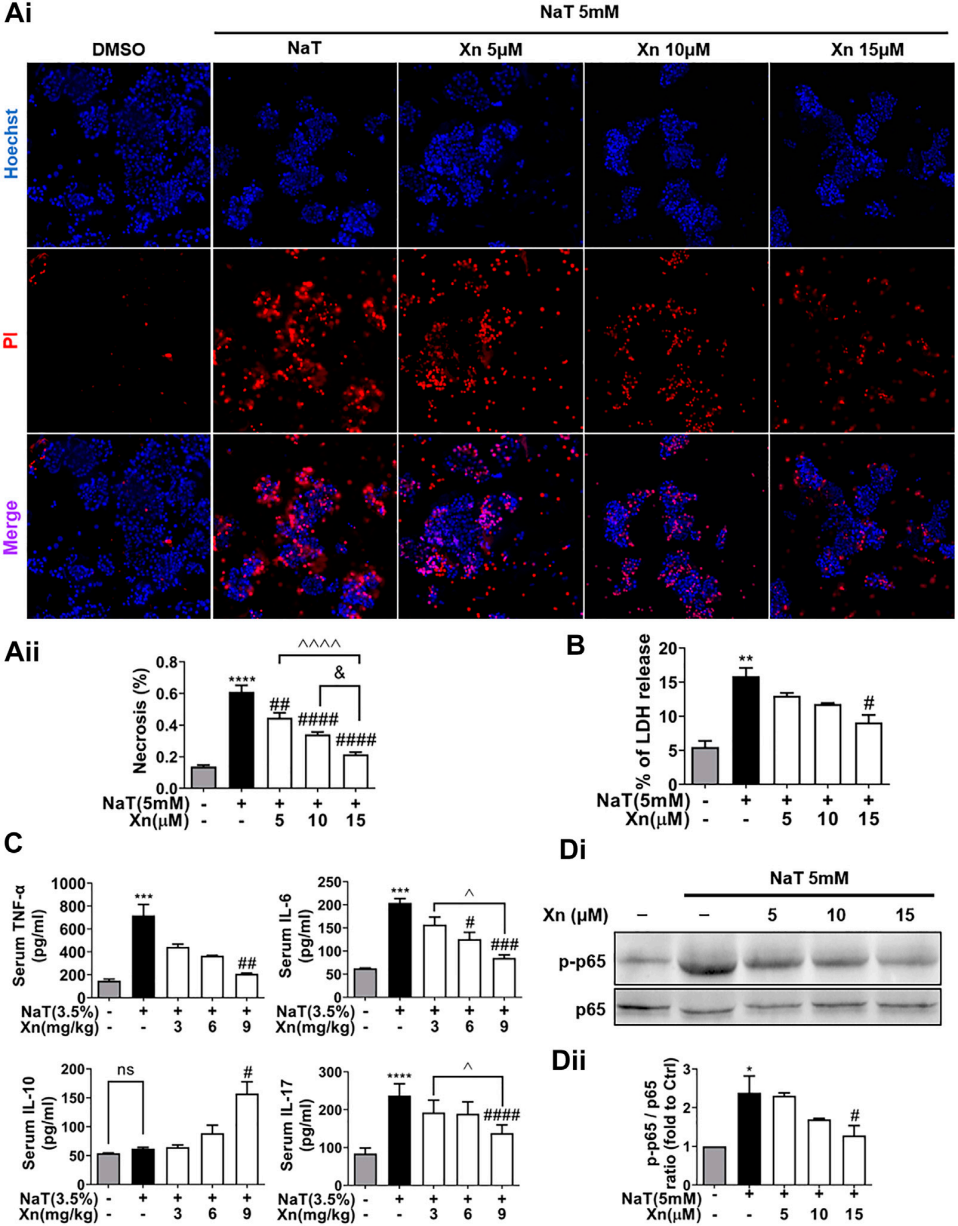
Xn concentration-dependently reduced NaT-induced necrosis and inflammation. **(Ai)** Pancreatic acinar cells staining with Hoechst 33342 (blue) and PI (red). **(Aii)** Necrosis rate quantification. **(B)** LDH release in the lysate of acinar cells. **(C)** Serum IL-6, TNF-α, IL-10 and IL-17. **(Di, Dii)** The expression of phosphorylated-p65 and p65 protein in acinar cells. (mean ± SD; *n* = 3–5) ^∗^ *p* < 0.05, ^∗∗^ *p* < 0.05, ^∗∗∗^ *p* < 0.05 and ^∗∗∗∗^ *p* < 0.0001 *versus* Sham. ^#^ *p* < 0.05, ^##^ *p* < 0.01, ^###^ *p* < 0.001 and ^####^ *p* < 0.0001 *versus* NaT. ^∨^ *p* < 0.05 *versus* Xn (5 μM). ^&&&&^ *p* < 0.0001 *versus* Xn (10 μM). ^*p* < 0.05, and ^^^^*p* < 0.0001 *versus* Xn (15 μM).

Acinar necrosis triggers strong inflammatory responses (Wang et al., 2016). To test the effects of Xn on NaT-induced necrosis *in vitro*, we evaluated the necrosis rate by calculating the ratio of PI/Hoechst positive cells and measuring LDH release in the extracellular medium. Figures 2Ai, B displays Xn concentration-dependently reduced NaT-induced acinar necrosis rate and LDH release. Figure 2C shows that NaT-SAP increased pro-inflammatory cytokines, such as TNF-α, IL-6, and IL-17. However, Xn treatment alleviated these levels. NaT-SAP did not affect IL-10, a cytokine that combats inflammation (Sziksz et al., 2015), whereas Xn treatment increased it. And NF-κB regulates the inflammation-related genes, which is why the activation of this pathway is related to the development of systemic complications during SAP (Satoh et al., 1999). As presented in Figures 2Di, Dii, Xn significantly suppressed p65 phosphorylation in NaT-incubated acinar cells. Treatment with a high dose of Xn (9 mg/kg *in vivo* and 15 μM *in vitro*) showed the greatest inhibitory effects on acinar necrosis and systemic inflammation in NaT-SAP. Hence, the following experiments only use a high dose of Xn (9 mg/kg *in vivo* and 15 μM *in vitro*).

### 3.3 Xn treatment alleviated NaT-induced oxidative stress

**FIGURE 3:**
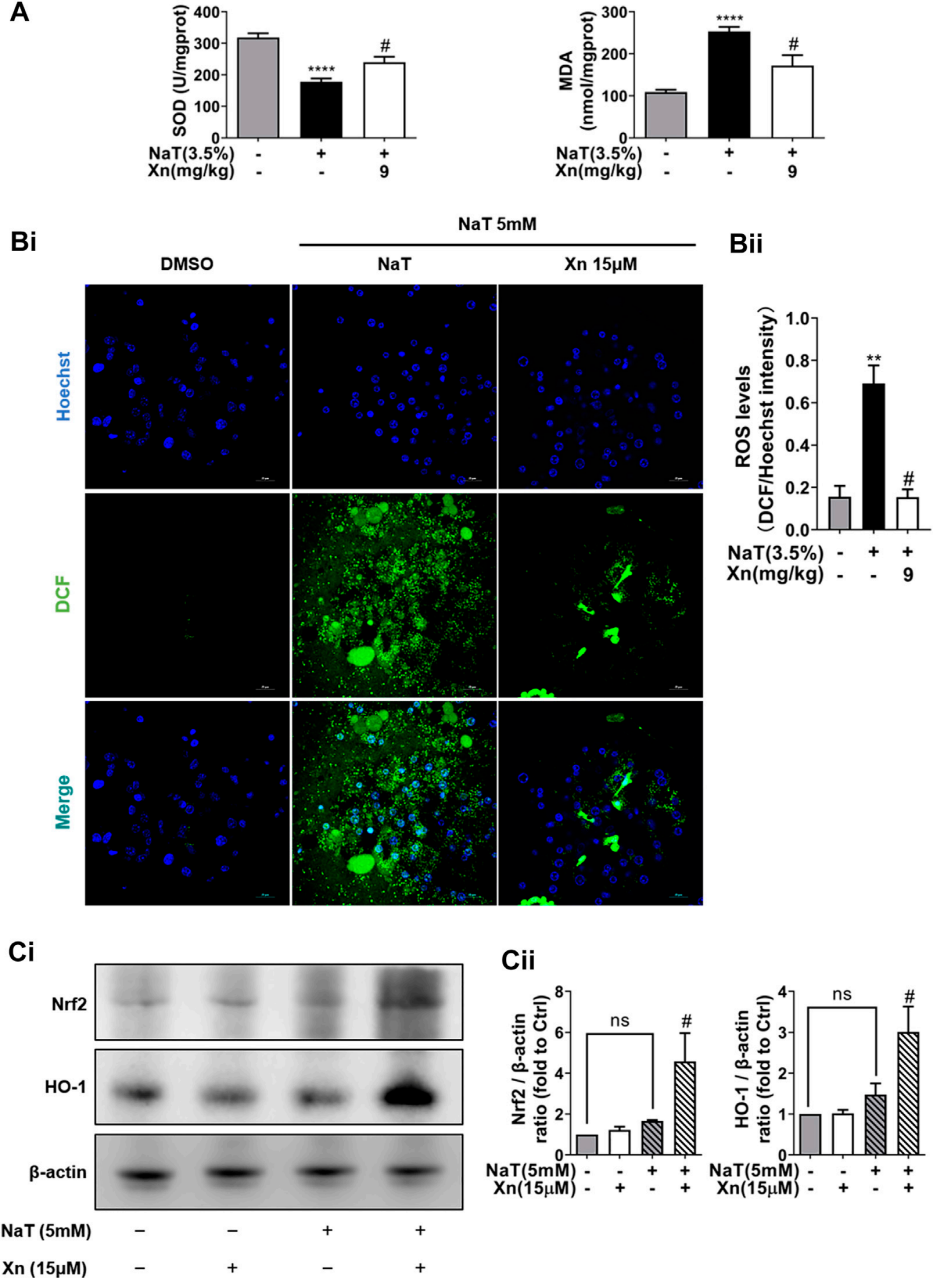
Xn treatment alleviated NaT-induced oxidative stress. **(A)** The pancreatic level of MDA and activity of SOD. **(Bi)** The fluorescence intensity of DCF (green) and Hoechst 33342 (blue) in primary acinar cells. **(Bii)** The Quantification of the fluorescence intensity. **(Ci)** Representative western blot of HO-1 and Nrf2. **(Cii)** HO-1 and Nrf2 protein quantification. (mean ± SD; *n* = 3–5). ^∗^ *p* < 0.05, ^****^ *p* < 0.00001 *versus* Sham, ^#^ *p* < 0.05 *versus* NaT.

SAP also relies heavily on oxidative stress, which is closely related to the inflammatory cascade (Habtezion et al., 2019). Then we detect the effect of Xn on oxidative stress in NaT-SAP. Figures 3Bi, Bii illustrates the large amount of ROS released by damaged pancreatic acinar cells after NaT treatment. However, Xn significantly inhibited cytosolic ROS release amounts. And results *in vivo* showed that NaT-SAP led to increased MDA level and decreased SOD activity. But Xn lessened SOD depletion and reduced MDA formation (Figure 3A). NaT-SAP did not alter the expression of antioxidative stress-related proteins Nrf2 and HO-1, but Xn increased their expression (Figures 3Ci, Cii). These findings provided treatment with Xn eased oxidative stress injury in SAP.

### 3.4 Xn treatment alleviated autophagy impairment in NaT-SAP

**FIGURE 4:**
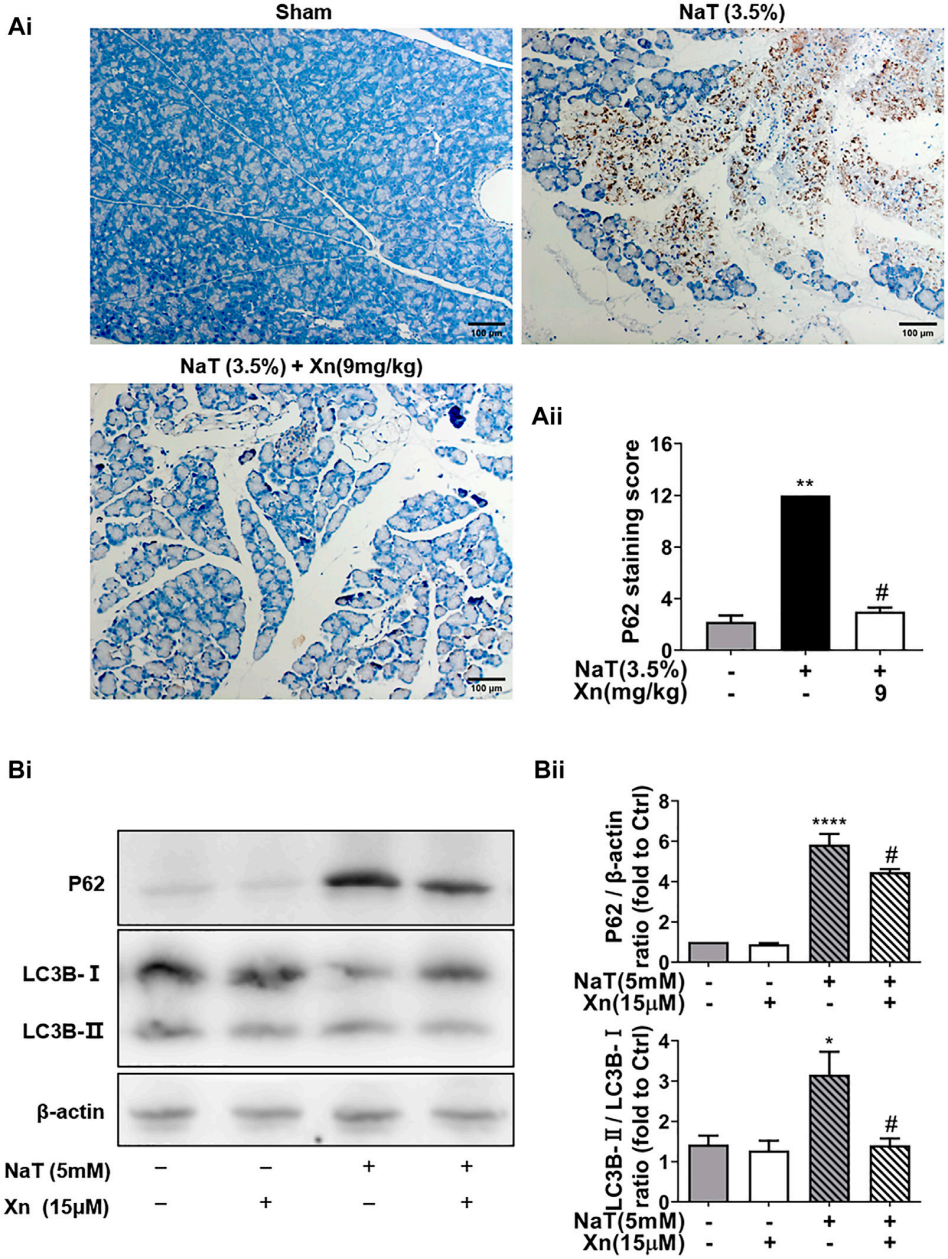
Xn treatment alleviated NaT-induced autophagy impairment. **(Ai)** Representative anti-p62 immunohistochemistry of pancreas sections from each group. **(Aii)** The p62 staining score levels. **(Bi)** Western blot of total protein levels of LC3, p62, and β-actin. **(Bii)** Quantification of LC3-II/LC3-I and p62 protein. (mean ± SD; *n* = 3–5) ^∗^ *p* < 0.05, ^****^ *p* < 0.00001 *versus* Sham, ^#^ *p* < 0.05 *versus* NaT.

The expanded inflammatory response in SAP can be triggered by dysregulated autophagy (Gukovskaya et al., 2017). The effect of Xn on autophagy in SAP was then investigated in order to understand how Xn exerts its protective effects. As one of the long-lived proteins, p62 is specifically degraded by autophagy. And its accumulation has been seen in pancreatitis models, indicating impaired autophagic flux (Gukovskaya et al., 2017). Immunohistochemistry was used for monitoring autophagy *in vivo*. NaT group showed more accumulation of p62 than Sham. Compared to the NaT group, the Xn group had less accumulation of p62 (Figures 4Ai, Aii). In addition, primary acinar cells incubated with NaT exhibited accumulations of p62 and the phosphatidylethanolamine conjugated LC3 (LC3B-II), while Xn downregulated the p62 and LC3B-II/LC3B-I ratio (Figures 4Bi, Bii). Those results suggest that Xn could restore the impaired pancreatic autophagy in NaT-SAP.

### 3.5 Xn enhanced autophagic flux *via* inhibiting the AKT/mTOR pathway

**FIGURE 5:**
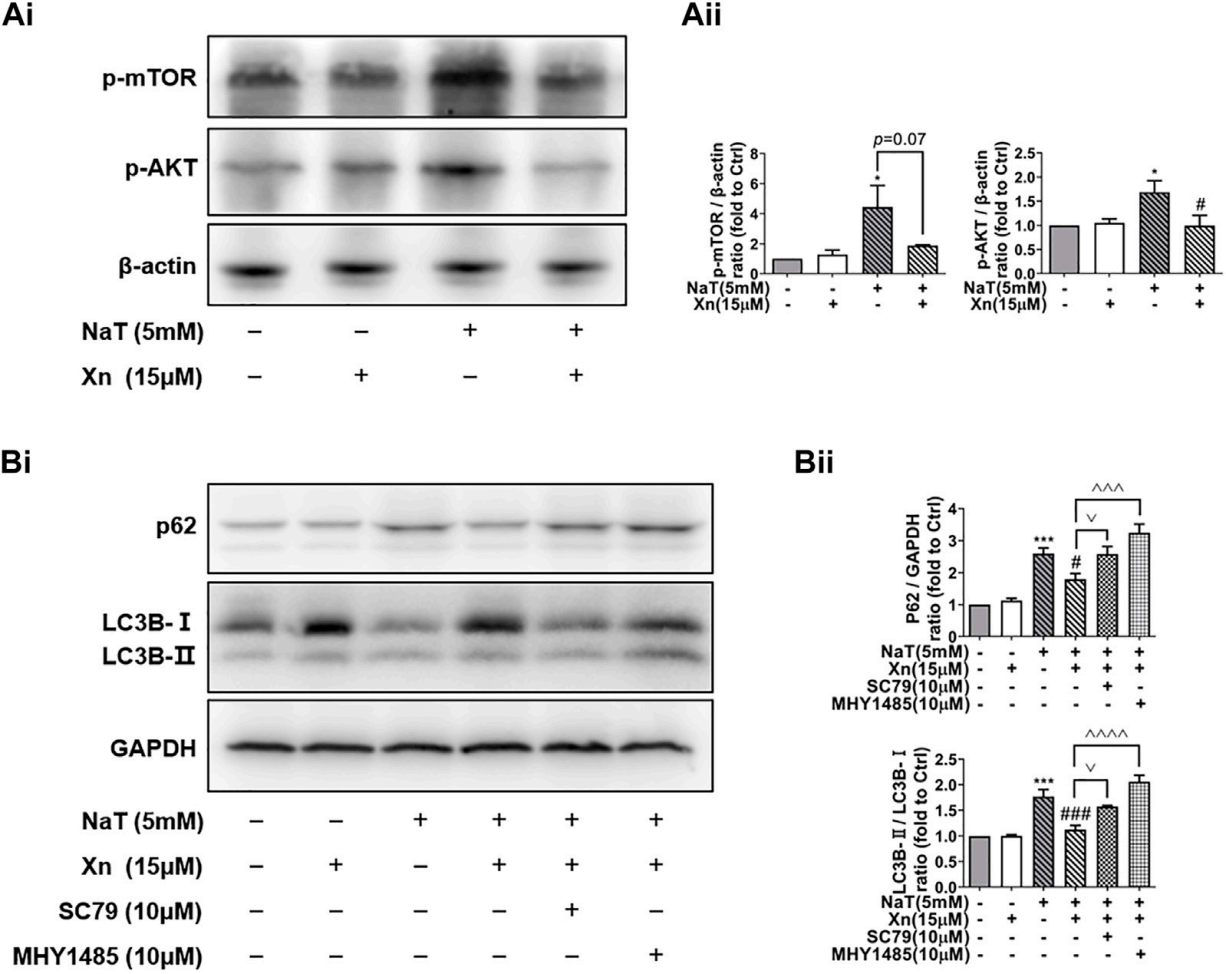
Xn enhanced autophagic flux by mTOR/AKT pathway inhibition. **(Ai)** Representative western blot of p-mTOR, p-AKT, and β-actin. **(Aii)** p-mTOR and p-AKT protein levels are quantified. **(Bi)** Representative western blot of LC3, p62, and GAPDH in acinar cells, measured after 50 min of incubation with NaT (5 μM) or Xn (15 μM) or AKT activator SC79 (10 μM) or mTOR activator MHY1485 (10 μM). (Bii) Analysis of the protein levels of p62 and the ratio of LC3-II to LC3-I. (mean ± SD; *n* = 3) ^*^ *p* < 0.05, ^***^ *p* < 0.001, ^****^ *p* < 0.0001 *versus* Sham; ^#^ *p* < 0.05, ^##^ *p* < 0.01, ^###^ *p* < 0.001, ^####^ *p* < 0.0001*versus* NaT. ^^*p* < 0.01, ^^^*p* < 0.001 and ^^^^*p* < 0.0001 *versus* MHY1485. ^∨^ *p* < 0.05 *versus* SC79.

Under nutrient and environmental stress, autophagy recycles macromolecules to provide energy and building blocks (Mizushima and Komatsu, 2011). AKT plays a critical role in regulating mTOR activity, which inhibits the initiation of autophagy, the formation of autophagosomes, and the transport of cell vesicles (Kim and Guan, 2015). According to our results, Phosphorylated-mTOR (p-mTOR) and phosphorylated-AKT (p-AKT) were enhanced by SAP, while Xn treatment downregulated both proteins (Figures 5Ai, Aii). In order to examine whether Xn regulates autophagy during SAP *via* the AKT/mTOR pathway, we co-incubated the pancreatic acinar cells after intervention with AKT activator SC79 or mTOR activator MHY1485. Activator groups exhibit significantly higher LC3B-II/LC3B-I ratios and accumulation of p62 than Xn groups, indicating that autophagic flux is inhibited (Figures 5Bi, Bii).

### 3.6 Xn inhibited inflammation and the oxidative stress partly by inhibition of AKT/mTOR

**FIGURE 6:**
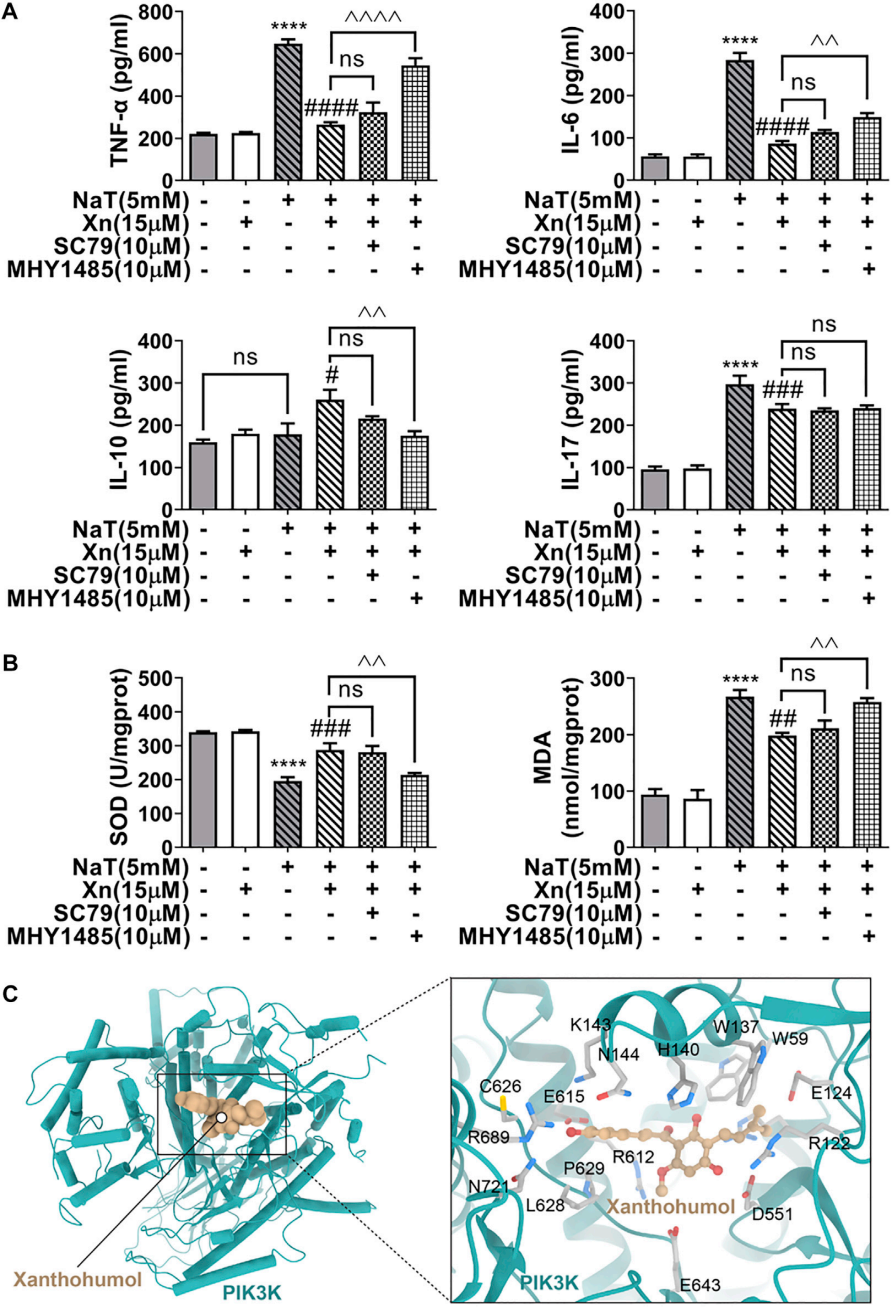
Xn inhibited inflammation and the oxidative stress in acinar cells partly *via* AKT/mTOR pathway. **(A)** The IL-6, IL-10, IL-17, and TNF-α levels are measured. **(B)** The MDA levels and SOD activities. **(C)** The representative visualized molecular binding models of Xn and PI3K. (mean ± SD; n = 3) ^*^ *p* < 0.05, ^***^ *p* < 0.001, ^****^ *p* < 0.0001 *versus* Sham; ^#^ *p* < 0.05, ^##^ *p* < 0.01, ^###^ *p* < 0.001, ^####^ *p* < 0.0001 *versus* NaT. ^^*p* < 0.01, ^^^*p* < 0.001 and ^^^^*p* < 0.0001 *versus* MHY1485. ^∨^ *p* < 0.05 *versus* SC79.

Deficiency in autophagy in SAP causes inflammation and oxidative stress (Antonucci et al., 2015). Researchers have reported cross-talk interactions among autophagy, oxidative stress, and inflammation (Li et al., 2015). In our study, treatment with Xn decreased TNF-α, IL-6, and IL-17, while increasing IL-10. Additionally, Xn reduced MDA formation and lessened SOD depletion. Activation of mTOR almost reversed the treatment effect of Xn on NaT-SAP induced inflammation and oxidative stress (Figures 6A, B). Interestingly, activation of mTOR did not change the IL-17 level (Figure 6A). Overall, Xn plays an anti-inflammatory and antioxidative stress role partly through inhibition of AKT/mTOR. And the underlying regulatory mechanism of Xn on IL-17 needs further study. In addition, a docking analysis was conducted to examine potential interactions and binding modes between the Xn and the PI3K (the upstream of the AKT/mTOR). And the representative visualized molecular binding models are shown in Figure 6C. Xn molecule had good binding affinity with the target PI3K.

## 4 Discussion

Natural products are promising for the treatment of SAP due to their safety and effectiveness (Yang et al., 2022). In this study, we innovatively found that Xn protected against NaT-SAP in mice. Xn considerably inhibited inflammation and rescued autophagy impairment during NaT-SAP. In addition, Xn enhanced autophagy flux *via* inhibition the AKT/mTOR. Notably, Xn might inhibit the AKT/mTOR pathway through inhibiting IL-17.

The pathogenesis of SAP involved inflammation, acinar cell necrosis, and oxidative stress (Gukovskaya et al., 2017; Saluja et al., 2019). SAP triggered an inflammatory cascade, leading to acute lung injury. Thus, early cytokine inhibition and reversal of distant organ injury are crucial in reducing SAP severity (Dawra et al., 2011; Ge et al., 2020). Xn has shown impressive anti-inflammatory and antioxidant properties, particularly in the case of osteoarthritis and acute lung injury in mice (Lv et al., 2017; Chen et al., 2021). In our study, the decreased serum amylase-lipase levels and pancreatic-lung pathological scores indicated that Xn protects mice from NaT-SAP. Furthermore, the findings *in vitro* also demonstrated that Xn inhibited acinar cell necrosis induced by NaT. Xn remarkably decreased acinar cell necrosis rate and LDH release in NaT-incubated acinar cells. Survival rates of rats with NaT-SAP have been improved by blocking NF-B activation (Satoh et al., 1999). In consistency with these findings, Xn treatment decreased the p65 protein phosphorylation to prevent an inflammatory response. Additionally, Xn has a protective effect against SAP induced acute lung injury, which is consistent with findings of Lv that Xn protects against LPS-induced acute lung injury through anti-inflammation and anti-oxidant (Lv et al., 2017). We speculated that Xn treated acute lung injury caused by SAP because it improved pancreatic injury. It is also possible that Xn directly alleviated lung injury. In the future, we can further study the possible signaling pathway of Xn to improve SAP induced lung injury. Therefore, treatment with Xn could be a promising remedy for SAP, and its specific mechanism needs to be studied.

Growing evidence suggests that SAP relies heavily on autophagy, making targeting autophagy an effective therapeutic and preventative strategy. SAP can be treated with natural products such as saponins, emodins, and picroside II by targeting autophagy (Piao et al., 2017; Yu et al., 2018; Han and He, 2021). Autophagy can be triggered by ROS generation (Signorelli et al., 2019). Our previous research proved ROS impaired autophagy in the spleen stimulated by SAP through PI3K/AKT/mTOR pathway (Wen et al., 2022). Meanwhile, autophagy can alleviate oxidative damage by degrading or phagocytizing oxidative substances (Li et al., 2015). Autophagy and ROS can regulate one another. Antioxidant strategies are also required for SAP (Booth et al., 2011; Yu and Kim, 2014). Numerous studies have reported that Xn protects cells from oxidative stress (Lv et al., 2017; Li et al., 2018). Consistent with these studies, Xn significantly alleviated SAP-associated oxidative stress. ROS impaired autophagy by activating the classic AKT/mTOR pathway (Wen et al., 2022). A high level of mTOR activation inhibits autophagy initiation and autophagosome nucleation (Saxton and Sabatini, 2017). Through blocking the AKT/mTOR pathway, Xn promotes autophagy and reduces p62 and LC3 accumulation. After activating AKT/mTOR pathway, autophagy was impaired, which verified our results. Moreover, the molecular docking results also showed Xn had good binding affinity with the target PI3K (the upstream of AKT/mTOR). Xn treated SAP by improving autophagy, anti-oxidative stress and anti-inflammation, which is consistent with previous findings (Han and He, 2021; Wen et al., 2022). Differently, ROS is the upstream of SAP induced splenic injury in study of Wen. Picroside II controlled the autophagic activity by affecting the NF-κB (Han and He, 2021). However, our study demonstrated that Xn may reduce oxidative stress by improving autophagy.

Pathogenesis of SAP is heavily influenced by cytokines, which induce systemic inflammation, tissue damage, and organ dysfunction. Inflammatory responses in the pancreas can be promoted by dysregulated autophagy (Gukovskaya et al., 2017). TNF-α is an early-onset pro-inflammatory cytokine that directly damages cells in multiple organs, causing necrosis, inflammation, and edema (Silva-Vaz et al., 2020). IL-6, as an early biomarker of severe organ failure and death, produces broad pro-inflammatory effects and leads to tissue damage (Zhang et al., 2013). IL-17 affects immune responses and interacts with inflammatory mediators in SAP-related microenvironments (Li et al., 2021). IL-10, a cytokine that combats inflammation, reduces inflammation by inhibiting cytokine production like TNF-α (Sziksz et al., 2015; Palathingal Bava et al., 2022). Studies have confirmed that the administration of therapeutic drugs would protect SAP by attenuating the pro-inflammatory cytokine and recovering the IL-10 level, which is in line with our study (Wang et al., 2011; Silva-Vaz et al., 2020; Li et al., 2021; Palathingal Bava et al., 2022). Further, when inhibiting autophagy, the anti-inflammatory effect of Xn was partially lost. Unexpectedly, inhibiting autophagy did not reverse the effect of Xn on IL-17. Interestingly, the effects of IL-17 on autophagy during SAP pathogenesis are unknown. A recent report has confirmed that IL-17 stimulated keratinocytes activated PI3K/AKT/mTOR signaling in psoriasis (Varshney and Saini, 2018). Based on these studies, our study found that IL-17 may be upstream of autophagy in SAP. Xn may inhibit the AKT/mTOR pathway by inhibiting IL-17.

However, the study still has some limitations. Firstly, the mechanism of Xn ameliorating SAP-induced acute lung injury has not been studied. In the future, we can further study the possible signaling pathway of Xn to improve SAP induced lung injury. Secondly, the mechanism of Xn on IL-17 needs further research. Also, gene silencing techniques and pharmacological inhibitors should be used to further investigate how autophagy and the IL-17 signaling pathway interact.

## 5 Conclusion

To summarize, we innovatively investigated the efficacy of Xn on pancreatic inflammation, oxidative stress, and autophagy. Further, Xn recovered autophagy flux in NaT-SAP *via* inhibiting AKT/mTOR pathway. According to these findings, Xn might become a potential therapeutic candidate for SAP, and autophagy might be one of its primary targets.

## Data Availability

The original contributions presented in the study are included in the article/supplementary materials, further inquiries can be directed to the corresponding authors.
